# IL-18 receptor axis in allergic conjunctivitis: a multi-omics study

**DOI:** 10.3389/fimmu.2025.1704591

**Published:** 2025-12-16

**Authors:** Liyun Yuan, Wangming Su, Liangpin Li, Yang Xiang, Zijiao Xu, Yuchuan Wang, Xia Hua, Xiaoyong Yuan

**Affiliations:** 1 School of Medicine, Nankai University, Tianjin, China; 2 Tianjin Key Laboratory of Ophthalmology and Visual Science, Tianjin Eye Institute, Tianjin Eye Hospital, Tianjin, China; 3 Ophthalmology Department, The Second Hospital of Longyan, Longyan, Fujian, China; 4 Aier Eye Institute, Changsha, China; 5 Aier Eye Hospital, Tianjin University, Tianjin, China

**Keywords:** allergic conjunctivitis, single-cell RNA sequencing, genome-wide association study, mucosal immunity, IL-18 receptor axis

## Abstract

**Purpose:**

The pathophysiology of allergic conjunctivitis (AC) is not fully explained by the traditional Th2-centric model. This study aimed to identify AC-associated candidate genes and delineate immune pathways, with a focus on cell-type–specific mechanisms that could contribute to disease heterogeneity.

**Methods:**

We employed an integrative multi-omics strategy, using three genomic analyses (FUSION, MAGMA, UTMOST) on human GWAS data to identify AC-associated candidate genes. These candidates were then investigated using single-cell RNA sequencing data from a mouse model of AC to map immune cell communication and signaling dynamics. Key pathways were validated in an independent ovalbumin-induced AC mouse model using clinical scoring, qRT-PCR and Western blot analysis.

**Results:**

This approach identified nine high-confidence AC-associated candidate genes, including key components of the IL-1 and Toll-like receptor families. In the AC mouse model, the IL-18 receptor components *Il18r1* and *Il18rap* were selectively upregulated in NK and T cells from allergic versus control mice and correlated positively with interferon-γ (*Ifng*) expression. Cell–cell communication and pseudotime analyses indicated an allergic-state network characterized by enhanced NK cell–linked IFN-γ signaling to antigen-presenting cells and dynamic changes in NF-κB and JAK–STAT pathway activity. In the OVA-induced model, conjunctival IL-18, IL-18R1, IL-18RAP and phosphorylated NF-κB p65 were increased in AC versus controls and showed a stepwise rise across mild, moderate and severe clinical groups.

**Conclusions:**

Across human genetics, single-cell transcriptomics and *in vivo* validation, an IL-18 receptor/IFN-γ axis in NK and T cells emerges as a reproducible module associated with AC and its severity. These findings extend the immunopathological framework of AC beyond a purely Th2-driven process and nominate IL-18R-linked signaling as a candidate pathway for future mechanistic and therapeutic studies.

## 1 Introduction

Allergic conjunctivitis (AC) represents a significant global health challenge, affecting up to 40% of some populations and severely impairing quality of life through symptoms like ocular itching, tearing, hyperemia, and edema (1, 2). The classic understanding of AC is rooted in a Type I IgE-mediated hypersensitivity reaction, which unfolds in a two-phase process (3, 4). The immediate phase is triggered upon allergen re-exposure, causing IgE-sensitized conjunctival mast cells to degranulate and release a cascade of inflammatory mediators like histamine. This is followed by a late-phase reaction, characterized by the infiltration of inflammatory leukocytes, most notably eosinophils, which perpetuate and amplify the allergic response through the release of cytotoxic proteins and other pro-inflammatory factors.

This entire cascade is traditionally seen as being orchestrated by a dominant Type 2 helper T cell (Th2) response and its signature cytokines (5). Interleukin-4 (IL-4) and IL-13 are crucial for driving B cells to produce allergen-specific IgE, while IL-5 is the primary cytokine responsible for the development, recruitment, and activation of eosinophils (6, 7). This well-defined IgE-Th2-mast cell-eosinophil axis underpins the majority of current AC therapies, including antihistamines, mast cell stabilizers, and corticosteroids (8). However, the frequent observation of suboptimal patient responses and the significant clinical heterogeneity of AC—spanning from mild forms like Seasonal Allergic Conjunctivitis (SAC) and Perennial Allergic Conjunctivitis (PAC) to severe, sight-threatening conditions like Vernal Keratoconjunctivitis (VKC) and Atopic Keratoconjunctivitis (AKC)—strongly suggest that this model is incomplete (9, 10).

The limitations of the Th2-centric paradigm highlight a critical need to explore non-canonical pathways. Emerging evidence implicates other immune cells, such as natural killer (NK) cells, in AC pathogenesis (11). Moreover, the role of the Th1 cytokine interferon-γ (IFN-γ) appears more complex than previously thought, with studies reporting its elevation in severe forms of AC, challenging the simplified Th1/Th2 antagonism model (12). Concurrently, genome-wide association studies (GWAS) have identified multiple genetic loci for AC and related atopic diseases. Studies of asthma, allergic rhinitis and atopic dermatitis have consistently implicated risk variants at *IL1RL1–IL18R1*, *TSLP* and *HLA class II* loci, indicating that these genes represent shared susceptibility hubs across allergic diseases (13–16). More recently, large biobank-based cohorts such as FinnGen have enabled AC-specific GWAS and TWAS analyses, further supporting a substantial overlap between ocular allergy and systemic atopic traits at the genetic level (17, 18).Yet, a major knowledge gap persists: how these genetic variations translate into specific cellular dysfunctions within the unique immune microenvironment of the ocular surface remains largely unverified (19, 20). Understanding this “gene-to-cell-to-pathway” mechanism is essential for moving beyond our current therapeutic plateau.

Therefore, this study was designed to address these critical gaps by employing an integrative, multi-omics strategy. We hypothesized that specific genetic susceptibility factors drive AC by regulating the conjunctival immune cell communication network. By linking human genetic data to validation in a disease-relevant animal model, we aimed to identify novel pathogenic axes beyond the classical Th2 paradigm, thereby providing a new molecular basis for understanding AC’s complexity and revealing promising new targets for future therapies.

## 2 Materials and methods

**Figure 1:**
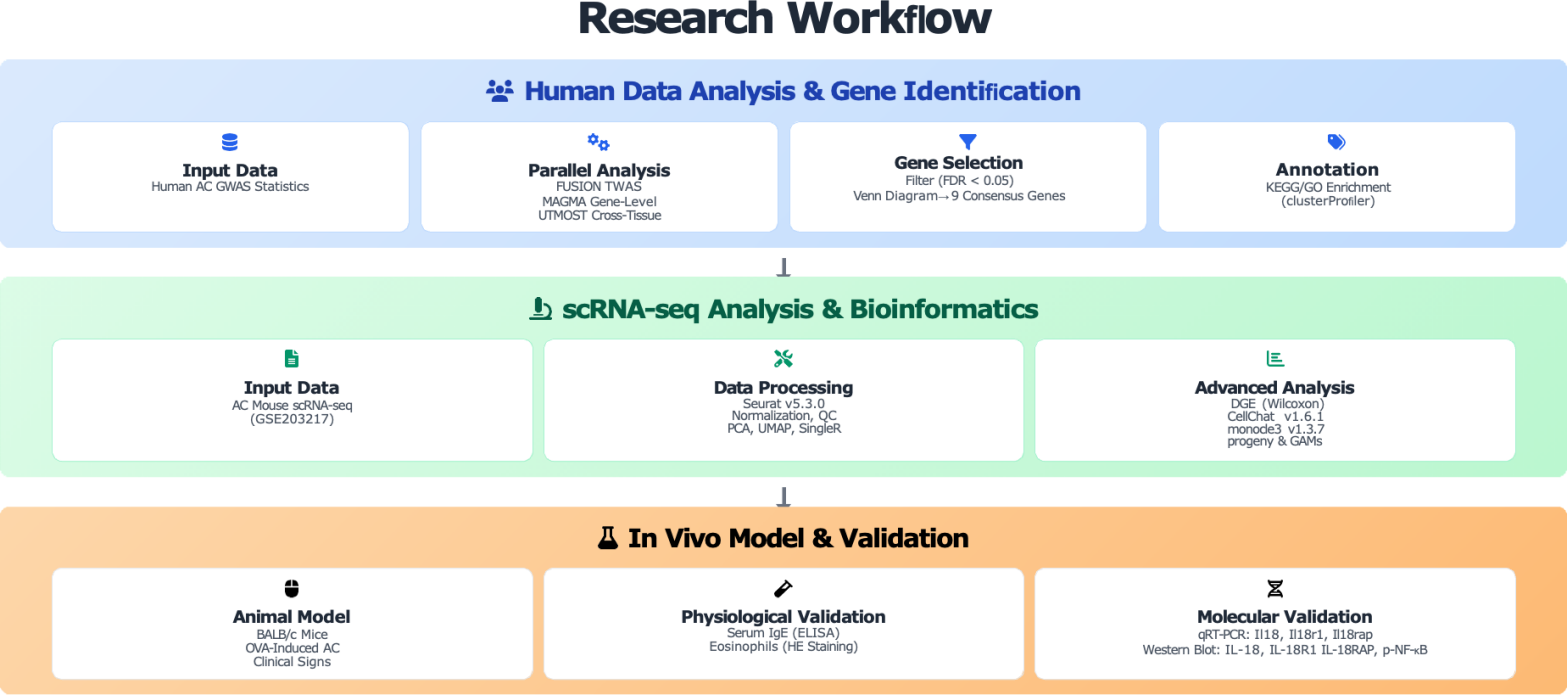
Research workflow: human multi-omics discovery, single-cell validation, and mouse model verification.

The overall workflow of this study is shown in **Figure 1**. In brief, the study comprised three stages (1): human genetic analyses to identify candidate susceptibility genes for allergic conjunctivitis (AC); (2) in-depth bioinformatic re-analysis of a single-cell RNA sequencing (scRNA-seq) dataset from an AC mouse model; and (3) *in vivo* OVA-induced AC modeling with molecular validation of key pathways.

### 2.1 Identification of candidate susceptibility genes for human AC

#### 2.1.1 Multi-method genetic association analysis

Publicly available genome-wide association study (GWAS) summary statistics for allergic conjunctivitis (endpoint H7_ALLERGICCONJUNCTIVITIS) were obtained from the FinnGen consortium (Release 11, R11; Kurki MI et al., Nature 2023; 613:508–518) (21). FinnGen integrates genotype data from a nationwide network of Finnish biobanks with longitudinal electronic health records from national registries.

According to the FinnGen R11 manifest (Data Freeze 13), the GWAS for H7_ALLERGICCONJUNCTIVITIS included 26,125 registry-based cases (19,877 female, 6,248 male) and 427,608 controls, for a total of 453,733 genotyped participants. Cases and controls were defined based on registry diagnoses following the FinnGen R11 clinical endpoint documentation. H7_ALLERGICCONJUNCTIVITIS is defined by ICD-10 codes H10.1, H10.4 and L20.8 and incorporates the sub-endpoints H7_CONJUNCTIVITISATOPIC (ICD-10 H10.1, L20.8; ICD-9 37200) and H7_USEOFEYEANTIALLERGENS (ATC S01GX). Controls were all genotyped individuals not meeting the endpoint definition after standard FinnGen quality control.

All analyses were conducted on summary-level GWAS data and thus identify statistical associations rather than causal effects. Mendelian randomization was not performed; formal causal inference is therefore beyond the scope of this study. FinnGen R11 summary statistics for H7_ALLERGICCONJUNCTIVITIS are publicly accessible from the FinnGen data repository (21).

MAGMA. Gene-level association analysis was performed using MAGMA v1.10 (22). SNP-level P values were aggregated into gene-based Z-scores while accounting for linkage disequilibrium (LD) using the 1000 Genomes Phase 3 European reference panel. Gene annotation was based on Ensembl GRCh38. Multiple testing was controlled by the Benjamini–Hochberg false discovery rate (FDR). MAGMA tested 19,107 genes genome-wide and identified 247 significant genes at FDR < 0.05.

FUSION (TWAS). A transcritome-wide association study (TWAS) was conducted using the FUSION software package (v3.0) (23) to link genetically predicted gene expression with allergic conjunctivitis risk. Because conjunctival eQTL data are unavailable, we used two complementary expression reference panels. First, the GTEx v8 multi-tissue panel (24) was used to capture shared systemic immune and epithelial regulatory effects relevant to allergic conjunctivitis. Second, a retina-specific panel derived from Ratnapriya et al. (25)was used as the only available large-scale ocular eQTL resource and as an eye-relevant surrogate for cis-regulatory variation in ocular tissues, including the ocular surface. Across both panels, FUSION tested 28,958 unique genes, including 28,372 from GTEx v8 (49 tissues) and an additional 586 retina-specific genes, using the 1000 Genomes European LD reference. For each gene, the model showing the stronger statistical evidence (larger |Z| and smaller P/FDR) was retained, yielding 427 significant genes at FDR < 0.05.

UTMOST. A cross-tissue TWAS was performed using the UTMOST model (26). which integrates expression quantitative trait loci (eQTL) data from 44 GTEx v8 tissues to detect shared regulatory effects across tissues. A Benjamini–Hochberg FDR < 0.05 threshold identified 35 significant genes among 3,750 tested.

#### 2.1.2 Functional annotation and homolog mapping of consensus genes

The intersection of significant genes from all three methods yielded nine consensus genes. These genes underwent functional enrichment analysis for KEGG pathways and gene ontology (GO) biological processes using the clusterProfiler R package (Padj < 0.05) (v4.16.0) (27). Mouse homologs of human candidate genes were identified using the biomaRt R package (v2.64.0) and Ensembl Genes database (version 108, GRCh38.p13) (28). Genes lacking mouse homologs were excluded.

### 2.2 scRNA-seq analysis

#### 2.2.1 scRNA-seq data processing and clustering

Publicly available single-cell RNA sequencing (scRNA-seq) data from the mouse model of allergic conjunctivitis (GSE203217) were reanalyzed (29). CD45^+^ conjunctival immune cells were pooled from 13 saline-treated and 11 HDM-sensitized mice. Raw 10x Genomics Chromium Single Cell 3′v3 count matrices for control and allergic libraries were downloaded separately, imported into Seurat v4.3.0, and merged into a single object (3,913 droplets). After quality control (cells with <200 or >2,500 detected genes, >10% mitochondrial transcripts or >50,000 unique molecular identifiers [UMIs] were excluded), 2,014 cells remained (758 control and 1,256 allergic), with a median of 4,082 UMIs and 1,560 detected genes per cell. Libraries were sequenced on an Illumina NovaSeq 6000, and CD45^+^ cells from multiple mice per group were processed as pooled samples and deposited as a single combined matrix; accordingly, GSE203217 was treated as one batch without additional batch correction.

Expression values were normalized using Seurat’s log-normalization (scale factor = 10,000). The 2,000 most variable genes were used for principal component analysis (PCA), and the first 10 principal components defined a shared nearest-neighbor graph. Louvain clustering was run over resolutions 0.1–1.0, and cluster stability was assessed with clustree; a resolution of 0.4 (10 clusters) provided a balance between under- and over-clustering and was used for downstream analyses. Uniform manifold approximation and projection (UMAP) was used for visualization.

Clusters were annotated into nine major immune and stromal cell types—T cells, NK cells, B cells, monocytes, macrophages, granulocytes, dendritic cells, fibroblasts and endothelial cells—based on clustering structure and canonical marker expression consistent with known conjunctival populations. For example, NK cells were defined by expression of *Ncr1*, *Nkg*7 and *Gzmb*, whereas T cells were defined by *Cd3d*, *Cd4* and *Cd8a*.

#### 2.2.2 Advanced bioinformatic analyses

*CellChat* (v1.6.1) analysis was performed separately by group, using mouse ligand-receptor databases (30). Overexpressed genes and interactions were identified, followed by communication probability and network aggregation analyses. IFN-II, CD80, and MHC-II pathways were prioritized in the comparative analysis.

Pseudotime trajectories for NK and T cells were reconstructed using Monocle3 (v1.3.7). In total, 162 NK cells and 776 T cells were included, and Ncr1^+^ NK cells were specified as the trajectory root to represent the earliest innate lymphocyte compartment in the allergic conjunctivitis model. NF-κB and JAK–STAT pathway activities were inferred with PROGENy (v1.30.0) using the default mouse footprint weight matrix (no custom re-weighting), and the resulting pathway scores were used directly for downstream modelling. Temporal dynamics and cell type–specific effects along pseudotime were evaluated using generalized additive models (GAMs; mgcv v1.9.3).

### 2.3 Animal model and *in vivo* validation

#### 2.3.1 Allergic conjunctivitis animal model

Male BALB/c mice (6–8 weeks old, 20–25 g) mice were randomly assigned to a control group (Control, n = 21) or an ovalbumin-induced allergic conjunctivitis group (OVA, n = 33). OVA mice were sensitized by intraperitoneal (i.p.) injection of 100 µg ovalbumin emulsified with 1 mg aluminum hydroxide adjuvant in 200 µL sterile phosphate-buffered saline (PBS) on days 0, 4, 7 and 13, whereas Control mice received i.p. injections of alum in PBS. From days 14 to 21, OVA mice were challenged once daily with bilateral topical instillation of 5 µL per eye of PBS containing 10 mg/mL OVA, while Control mice received topical PBS alone.

Clinical evaluation: After the final topical challenge (day 21), clinical signs of allergic conjunctivitis were evaluated by a trained observer masked to group assignment. Clinical scoring followed the scheme originally described for murine experimental allergic conjunctivitis (31) Chemosis, conjunctival redness, lid edema and tearing/discharge were each graded on a 0–3 scale (0 = absent, 1 = mild, 2 = moderate, 3 = severe), and the four scores were summed to obtain a total clinical score ranging from 0 to 12, with higher scores indicating more severe disease.

**Figure 2:**
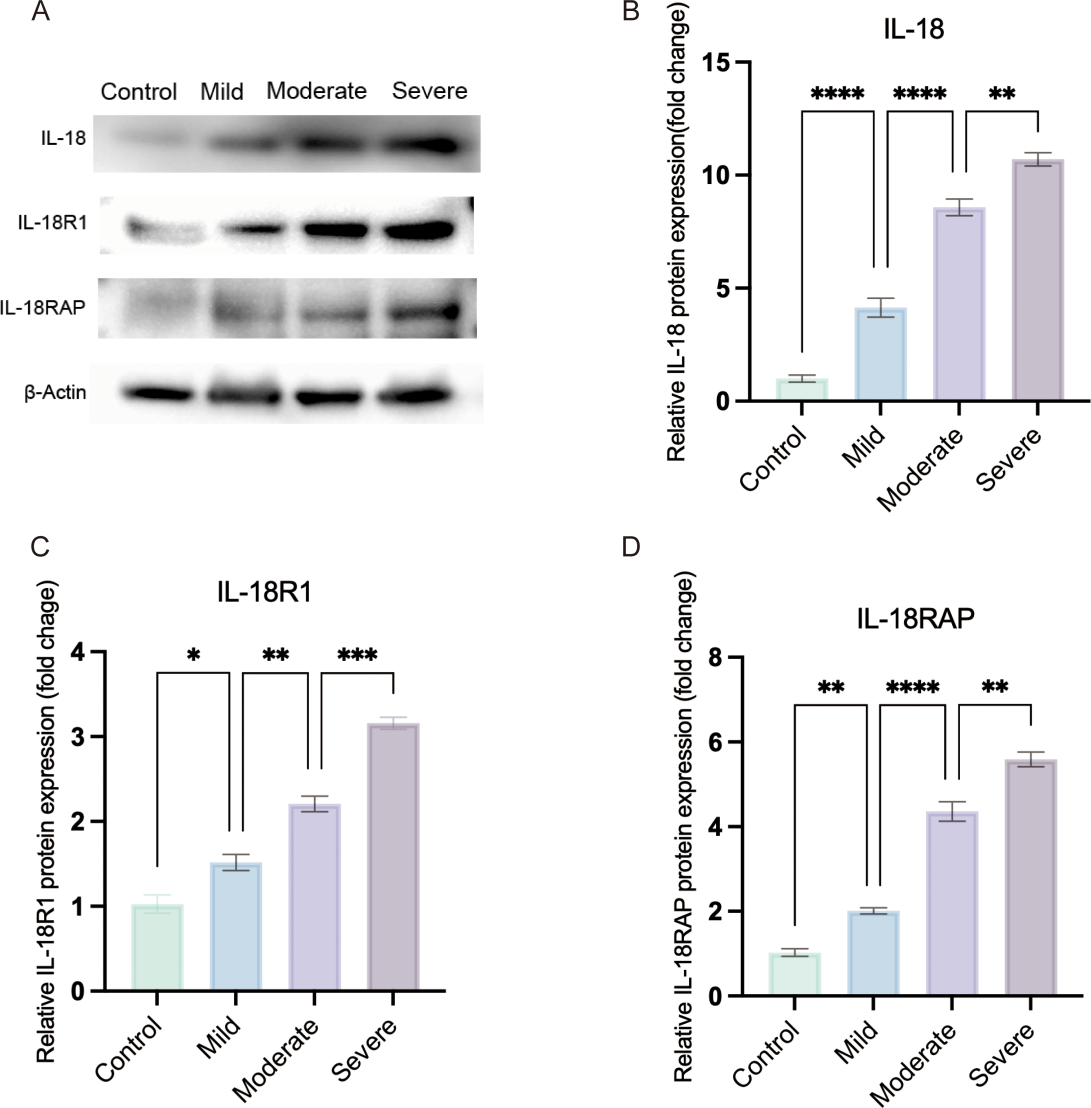
IL-18 receptor axis expression increases with clinical severity in OVA-induced allergic conjunctivitis. **(A)** Representative western blots of conjunctival IL-18, IL-18R1 and IL-18RAP protein levels in Control, Mild, Moderate and Severe OVA-induced AC mice; β-actin was used as a loading control. **(B–D)** Densitometric quantification of IL-18 **(B)**, IL-18R1 **(C)** and IL-18RAP **(D)** expression normalized to β-actin and expressed relative to the control group. OVA-challenged mice were stratified into mild, moderate and severe groups according to clinical symptom scores. Statistical analysis was performed using ordinary one-way ANOVA with Tukey’s multiple-comparison test; linear trend across ordered severity categories was assessed using the test for linear trend in Prism. (*P < 0.05, **P < 0.01, ***P < 0.001, ****P < 0.0001).).

**Figure 3:**
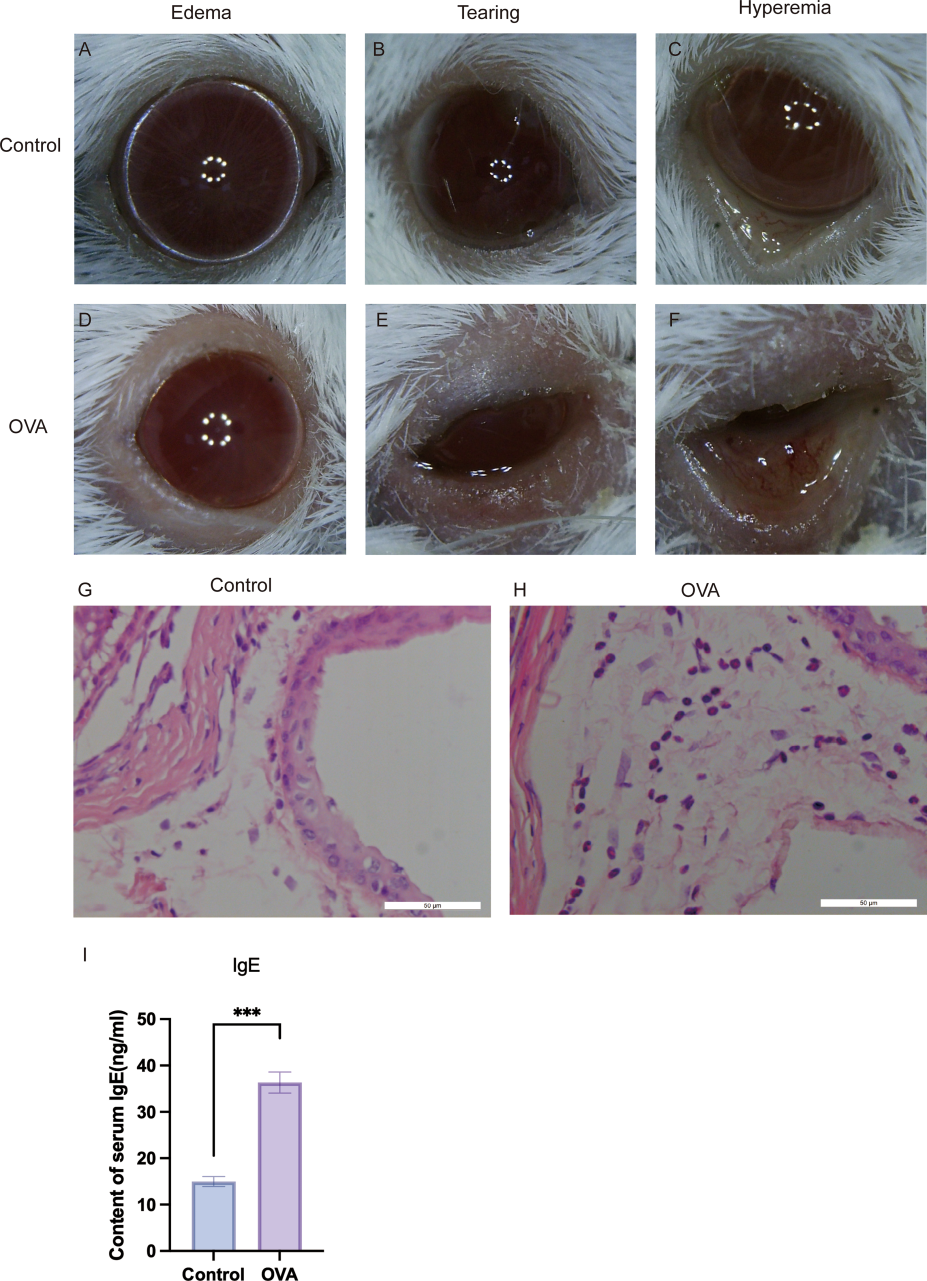
Validation of the ovalbumin (OVA)-induced allergic conjunctivitis (AC) mouse model. **(A–F)** Representative macroscopic photographs showing the clinical signs in Control and OVA-sensitized mice after the final challenge. Compared to the Control group **(A–C)**, mice in the OVA group **(D–F)** displayed hallmark symptoms of AC, including pronounced edema (eyelid swelling), tearing (ocular discharge), and hyperemia (conjunctival redness). **(G, H)** Representative photomicrographs of conjunctival tissue sections stained with Hematoxylin and Eosin (HE). The OVA group **(H)** shows substantial infiltration of inflammatory cells, predominantly eosinophils, within the conjunctival stroma, a feature absent in the Control group **(G)**. Scale bar = 50 µm. **(I)** Quantification of total serum immunoglobulin E (IgE) levels measured by ELISA. The OVA group exhibited significantly elevated levels of serum IgE compared to the Control group. ***P < 0.001.

**Figure 4:**
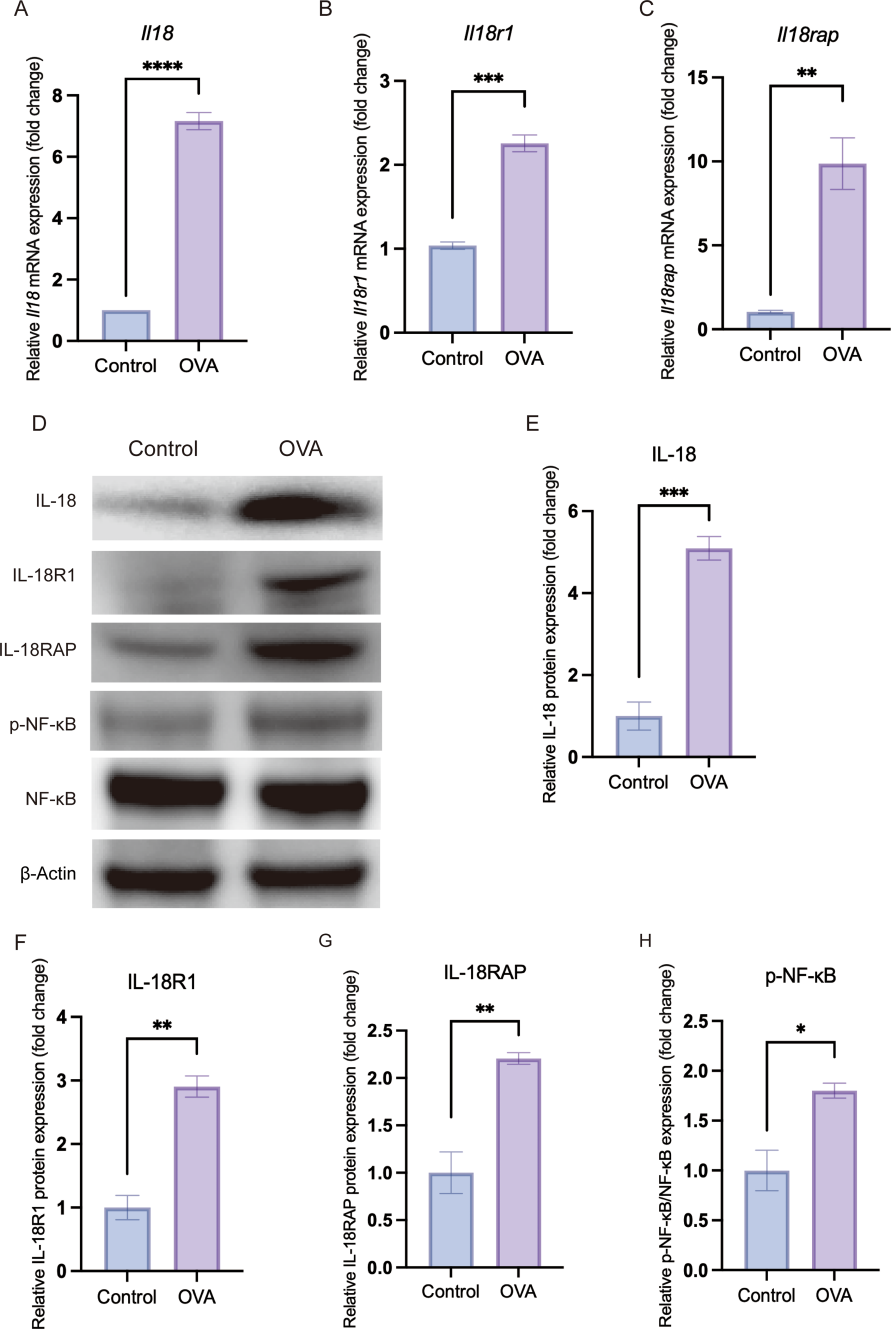
*In vivo* validation confirms upregulation of IL-18/IL-18 receptor axis and activation of the NF-κB pathway in allergic conjunctival tissue. Conjunctival tissues were harvested from Control and OVA-induced allergic conjunctivitis (OVA) mice. All graphical data are presented as mean ± SEM. (*P < 0.05, **P < 0.01, ***P < 0.001, ****P < 0.0001). **(A–C)** Relative mRNA expression levels of *Il18***(A)**, *Il18r1***(B)** and *Il18rap***(C)**, as determined by quantitative real-time PCR (qRT-PCR) and normalized to the endogenous control β-actin. **(D)** Representative Western blot images showing the protein levels of IL-18, IL-18R1, IL-18RAP, phosphorylated NF-κB p65 (p-NF-κB), total NF-κB p65 and the loading control β-actin in conjunctival lysates. **(E–H)** Densitometric quantification of Western blot bands showing a significant increase in IL-18 **(E)**, IL-18R1 **(F)** and IL-18RAP **(G)** protein expression, as well as an increased ratio of p-NF-κB to total NF-κB **(H)**, indicating heightened NF-κB pathway activation in the OVA group.

Subgroup stratification for severity analysis: For Western blot analysis of severity-dependent changes in IL-18 pathway activation (**Figure 2**), 18 OVA-challenged mice were selected from the AC cohort and ranked according to their total clinical scores. Based on the predefined score ranges derived from the established scoring system (31), these mice were stratified into three severity groups of equal size (n = 6 per group): mild (total score 1–4), moderate (5–8) and severe (9–12). For all other analyses (serum IgE, histology, qRT-PCR and the initial Western blot validations in **Figures 3**, **4**), AC mice were analyzed as a single group versus controls, with sample sizes reported in Sections 3.3–3.5.

#### 2.3.2 Histopathological examination of conjunctival tissue

Following euthanasia, conjunctival tissues were immediately dissected and fixed for 24 hours in 4% paraformaldehyde (PFA). The fixed tissues were subsequently dehydrated through a graded series of ethanol, cleared with xylene, and embedded in paraffin. Five-micrometer-thick sections were cut from the paraffin blocks and stained with Hematoxylin and Eosin (HE) according to standard protocols. The stained sections were then examined and photographed using a light microscope.

#### 2.3.3 Serum total IgE detection

Serum collected after clotting and centrifugation (600g, 10 min). Serum total IgE levels were measured in a subset of animals, including four control mice (Control, n = 4) and four OVA-challenged mice (OVA, n = 4), using a mouse IgE ELISA kit (Solarbio, CSEKM-0345) according to the manufacturer’s instructions. Each serum sample represented one mouse and was assayed in duplicate.

#### 2.3.4 Quantitative real-time PCR

For qRT-PCR analysis, bulbar and palpebral conjunctival tissues from both eyes of each mouse were carefully dissected and pooled per animal. Total RNA was extracted from conjunctival tissues of six Control mice (Control, n = 6) and six OVA-challenged mice (OVA, n = 6), representing a subset of the total OVA cohort, using an EZB-RN4 kit (EZBioscience) according to the manufacturer’s protocol. Within each group, tissues from two mice were combined to generate one biological replicate, yielding three pooled samples per group (n = 3 biological replicates per group, two mice per replicate).

RNA quantity and purity were assessed with a NanoDrop spectrophotometer (Thermo Fisher Scientific), and only samples with an A260/A280 ratio between 1.8 and 2.0 were used for cDNA synthesis. One microgram of RNA was reverse transcribed using the PerfectStart^®^ Uni RT&qPCR Kit. RT-qPCR was performed using the kit’s PerfectStart^®^ Green qPCR SuperMix. The sequences of all primers used for this analysis are provided in **Table 1**. β-actin served as the endogenous control. Relative expression by 2-ΔΔCt method (32).

**Table 1:** Sequences of mouse primers used for qRT-PCR analysis.

*Il18*-Forward 5’-3’	GACTCTTGCGTCAACTTCAAGG
*Il18*-Reverse 5’-3’	CAGGCTGTCTTTTGTCAACGA
*Il18rap*-Forward 5’-3’	AGACTACTTCCTGAGCACAAGA
*Il18rap*-Reverse 5’-3’	TGTCCTTACCAATGGTTCTCACT
*Il18r1*-Forward 5’-3’	TCACCGATCACAAATTCATGTGG
*Il18r1*-Reverse 5’-3’	TGGTGGCTGTTTCATTCCTGT
*Actb*-Forward 5’-3’	GATTACTGCTCTGGCTCCTAGC
*Actb*-Reverse 5’-3’	GACTCATCGTACTCCTGCTTGC

#### 2.3.5 Western blot analysis

For protein analysis, bulbar and palpebral conjunctival tissues from both eyes of each mouse were dissected and pooled per animal, then homogenized in RIPA lysis buffer containing protease and phosphatase inhibitors. Lysates were cleared by centrifugation, and protein concentrations were determined using a BCA assay (Thermo Fisher Scientific).

For the initial validation of IL-18 pathway activation (**Figures 3**, **4**), conjunctival lysates from six Control mice and six OVA-challenged mice (OVA) were analyzed by Western blot. Within each group, tissues from two mice were pooled to generate one biological replicate, yielding three pooled samples per group (n = 3 biological replicates, two mice per replicate). For severity-stratified analyses of IL-18, IL-18R1 and IL-18RAP (**Figure 2**), conjunctival tissues from six Control mice and from six mice in each OVA severity subgroup (mild, moderate and severe) were processed in the same way, again yielding three pooled samples per group.

Equal amounts of protein from each pooled sample were separated by SDS-PAGE and transferred onto PVDF membranes. After blocking with 5% non-fat milk in TBST, membranes were incubated overnight at 4 °C with primary antibodies against IL-18 (ABMART), IL-18R1 (ABMART), IL-18RAP (ABMART), NF-κB p65 (Proteintech), phospho-NF-κB p65 (Proteintech) and β-actin (Proteintech), followed by appropriate HRP-conjugated secondary antibodies. Following incubation with an HRP-conjugated secondary antibody, signals were detected via enhanced chemiluminescence (ECL), and band densities were quantified using ImageJ software.

### 2.4 Statistical analysis

Unless otherwise noted, tests were two-sided (33). For MAGMA, FUSION, and UTMOST, multiple testing was controlled using the Benjamini–Hochberg false discovery rate (BH-FDR). For the pseudotime analyses, we fitted generalized additive models (GAMs; mgcv v1.9.3) with cell type as a factor and cell type–specific smooth functions of pseudotime. The interaction between pseudotime and cell type was assessed by comparing full and reduced models using ANOVA, and the resulting P values were BH-adjusted across the pre-specified pathway comparisons.

For the mouse validation experiments, comparisons between Control and OVA groups were analyzed using unpaired t-tests. Western blot data across the four severity groups (Control, Mild, Moderate and Severe) were analyzed by ordinary one-way ANOVA to test for overall group differences, followed by a test for linear trend across the ordered severity groups (GraphPad Prism v10).

### 2.5 Ethical statement

#### 2.5.1 Animal research

All animal experiments were conducted in strict accordance with the ARVO Statement for the Use of Animals in Ophthalmic and Vision Research and were approved by the Tianjin Nankai Hospital Animal Ethical and Welfare Committee (No. NKYY-DWLL-2021–043). Mice were euthanized by intraperitoneal injection of sodium pentobarbital (MedChemExpress) at 200 mg/kg (diluted in sterile saline); loss of pedal withdrawal reflex and cessation of respiration were verified, followed by cervical dislocation to confirm death. Procedures were conducted in line with the AVMA Guidelines for the Euthanasia of Animals (2020). All efforts were made to minimize animal suffering.

#### 2.5.2 Human data

This study utilized publicly available summary-level data from human GWAS. The original studies that generated this data had received appropriate institutional review board approval and obtained informed consent from all participants. Our secondary analysis of this anonymized, aggregated data did not require additional ethical approval.

## 3 Results

### 3.1 IL18R locus and a nine-gene innate immune signature underlie genetic risk for AC

**Figure 5:**
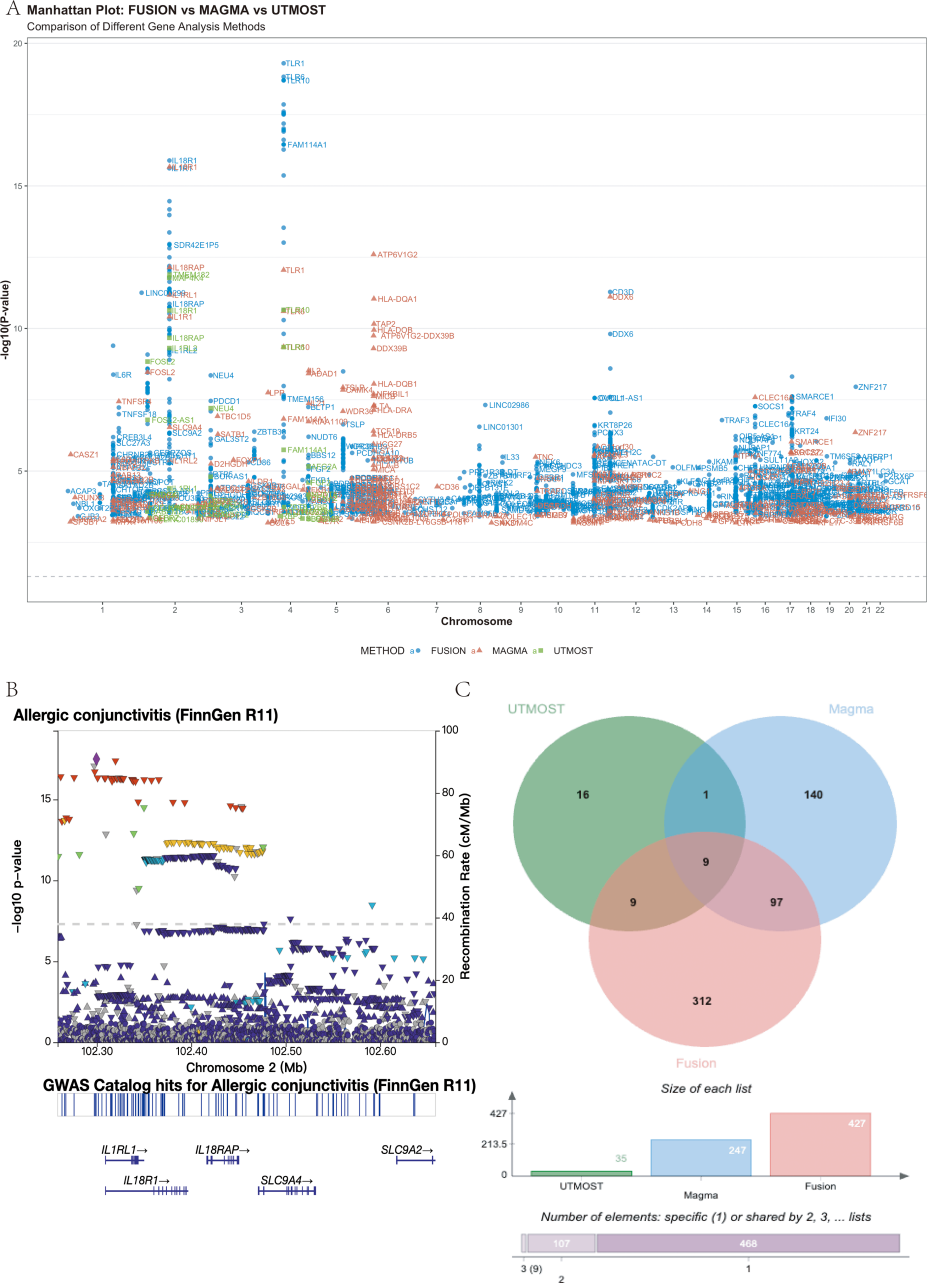
Multi-omics integration identifies a core set of nine potential risk genes for allergic conjunctivitis (AC). **(A)** Manhattan plot showing genomic locations (x-axis) and significance (−log_10_ P, y-axis) of AC-associated loci identified by FUSION (blue), MAGMA (red), and UTMOST (green). The dashed line indicates the *FDR < 0.05* threshold. Top loci, including *IL1RL1, TLR6, HLA-DQA1*, and *IL18R1/IL18RAP*, are labeled. **(B)** Regional association plot of the *IL18R1/IL18RAP* locus (chr2: 102.25–102.65 Mb, GRCh38) highlighting the lead SNP rs72823641 (*P* = 4.11 × 10^-^¹^8^). A single LD block spans both genes. **(C)** Venn diagram showing overlap among significant genes from the three analyses (*FDR < 0.05*), revealing nine shared candidates: *IL1RL2, IL1RL1, IL18R1, IL18RAP, GAL3ST2, TLR10, TLR6, BBS12, and FAM114A1.*.

Genome-wide association analysis of the FinnGen R11 cohort identified several loci reaching genome-wide significance (P < 5 × 10^-8^), including regions at *IL1RL1, TLR6, HLA-DQA1* and the *IL1RL1–IL18R1–IL18RAP* cluster (**Figure 5A**). At the 2q12 locus, regional association plots showed a single prominent peak spanning *IL18R1* and *IL18RAP* within one LD block (lead SNP rs72823641, P = 4.11 × 10^-^¹^8^; −log_10_P = 17.4), indicating a single genomic locus encompassing the IL18R gene cluster (**Figure 5B**). Variants proximal to *IL1RL1* displayed similar LD patterns to rs72823641, suggesting that the apparent IL1RL1 signal reflects the same association block rather than an independent locus.

To prioritize susceptibility genes, we integrated three gene-level association methods. FUSION, MAGMA and UTMOST identified 427, 247 and 35 significant genes, respectively (FDR < 0.05). Intersection of these results yielded nine high-confidence candidate genes common to all three approaches: *IL1RL2, IL1RL1, IL18R1, IL18RAP, GAL3ST2, TLR10, TLR6, BBS12* and *FAM114A1* (**Figure 5C**). Detailed per-gene statistics are provided in **Supplementary Table S1**.

**Figure 6:**
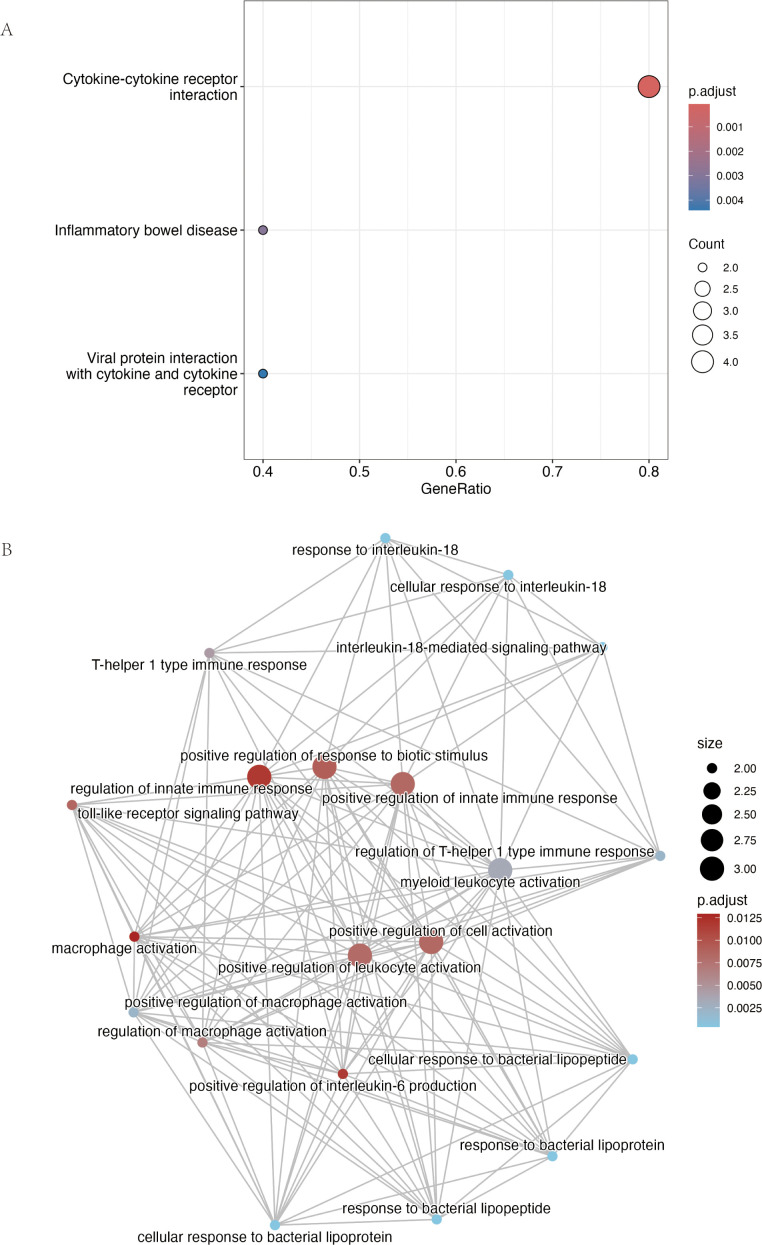
Functional enrichment analysis of the nine consensus risk genes highlights their roles in cytokine and innate immune signaling. **(A)** Dot plot summarizing the results of the Kyoto Encyclopedia of Genes and Genomes (KEGG) pathway enrichment analysis. The plot displays the most significantly enriched pathways. The size of each point is proportional to the number of consensus genes involved in the pathway (Count), while the color scale corresponds to the statistical significance (adjusted p-value). **(B)** Network visualization of enriched Gene Ontology Biological Process (GO-BP) terms generated using the R package clusterProfiler. Each node represents a distinct biological process and connecting lines (edges) indicate that the processes share associated genes. Node size is proportional to the number of genes within that term, and the color gradient reflects the adjusted p-value. The network reveals major functional clusters related to interleukin-18 signaling, Toll-like receptor pathways, and the regulation of innate immune responses such as macrophage activation.

Functional enrichment of these nine genes revealed strong immune involvement (**Figure 6**). KEGG analysis highlighted “Cytokine–cytokine receptor interaction” (4/9 genes, P-adj = 7.05 × 10^-5^), while GO terms included the “interleukin-18–mediated signaling pathway” (*IL18R1, IL18RAP*) and responses to bacterial components via *TLR6* and *TLR10*. Together, these data indicate that genetic risk for AC converges on an innate immune network dominated by IL-1 family and Toll-like receptor signaling.

### 3.2 IL-18 receptor components are selectively upregulated in allergic NK and T cells

In a public scRNA-seq dataset from an AC mouse model, the *Il18r1* homolog of the IL-18 receptor was significantly upregulated in conjunctival tissue of allergy-model mice compared with controls (avg_log2FC = 1.15, P-adj = 0.02657758), while *Il18rap* (the accessory protein) showed a trend towards increased expression (**Table 2**).

**Table 2:** Differential expression of *Il18r1* and *Il18rap* in the allergic conjunctivitis mouse model conjunctiva (scRNA-seq).

Gene	*p*-value (raw)	avg_log2FC	pct.1 (Allergy)	pct.2 (Control)	*p*-valueadj
*Il18r1*	8.558781 × 10^-7^	1.217	0.193	0.115	0.02657758
*Il18rap*	8.441956× 10^-3^	0.691	0.165	0.124	1.00000000

**Figure 7:**
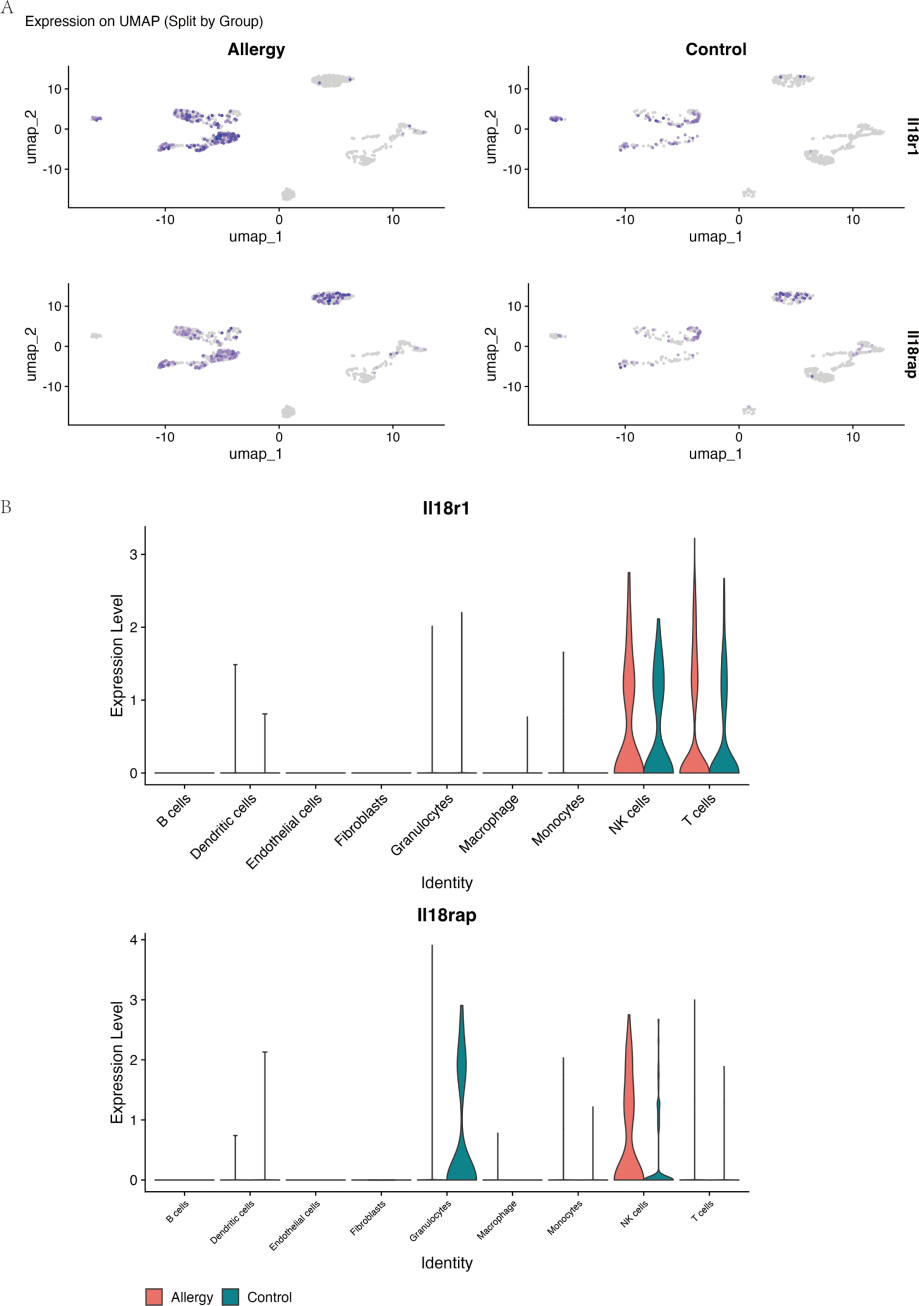
Il18r1 and Il18rap are selectively upregulated in NK and T cells during allergic conjunctival inflammation. Data are from single-cell RNA sequencing of conjunctival tissue from the allergic conjunctivitis (AC) mouse model. **(A)** UMAP-based feature plots showing the log-normalized expression of *Il18r1 (*top row) and *Il18rap* (bottom row) in single cells from Allergic (left) and Control (right) mice. The intensity of the purple color corresponds to the level of gene expression within each cell. **(B)** Violin plots comparing the expression levels of *Il18r1* and *Il18rap* across nine major cell types, separated by experimental group (Allergy, red; Control, teal). The plots demonstrate that expression of both IL-18 receptor components is largely restricted to NK cells and T cells and is substantially elevated in these specific cell populations in the Allergy group.

Cell type–resolved analysis showed that *Il18r1* and *Il18rap* expression was sparse in control mice but became markedly enriched in NK cells and T cells from allergic mice (**Figure 7**). These findings identify NK and T cells as the main cell populations in which IL-18 receptor signaling is amplified during allergic conjunctival inflammation.

### 3.3 The IL-18R/IFN-γ axis reinforces APC–T cell crosstalk in AC

**Figure 8:**
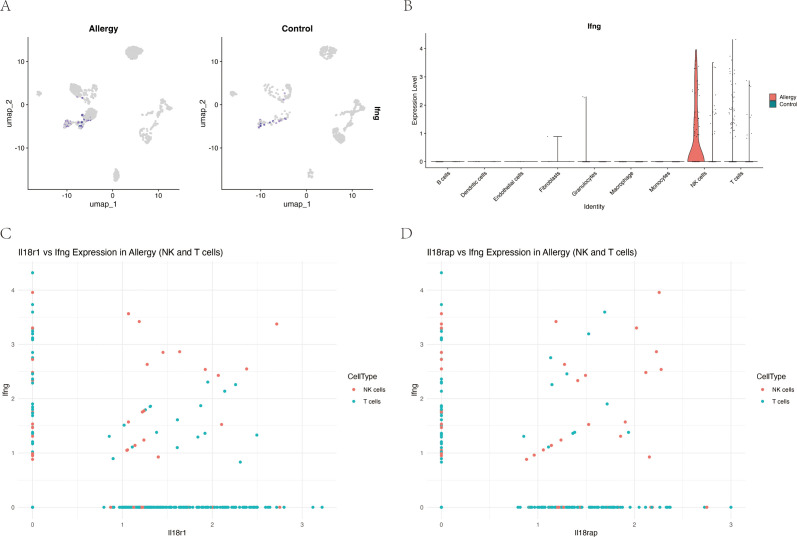
Expression of IL-18 receptor components correlates with interferon-γ (*Ifng*) expression in NK and T cells during allergic conjunctivitis. **(A)** UMAP feature plots showing log-normalized expression of *Ifng* in conjunctival cells, separated by Allergic and Control groups. **(B)** Violin plot quantifying *Ifng* expression across major cell types, highlighting its specific upregulation in NK cells and T cells from the Allergy group (red) compared to the Control group (teal). **(C)** Scatter plot showing a significant positive correlation between *Il18r1* and *Ifng* expression in single NK cells (red) and T cells (teal) from the Allergy group (Spearman’s ρ = 0.087, *P* = 0.024). **(D)** Scatter plot showing a stronger positive correlation between *Il18rap* and *Ifng* expression in the same cell populations (Spearman’s ρ = 0.137, *P* = 0.0003).

Given prior evidence that IL-18 can induce IFN-γ in other systems (34), we examined their relationship at the single-cell level in our model. In NK and T cells from allergic mice, Il18r1 expression showed a modest but significant positive correlation with *Ifng* (ρ = 0.087, *P* = 0.024), and *Il18rap* exhibited a somewhat stronger correlation (ρ = 0.137, *P* = 0.0003) (**Figure 8**), suggesting that higher IL-18 receptor expression is associated with increased IFN-γ transcription in these lymphocyte subsets.

**Figure 9:**
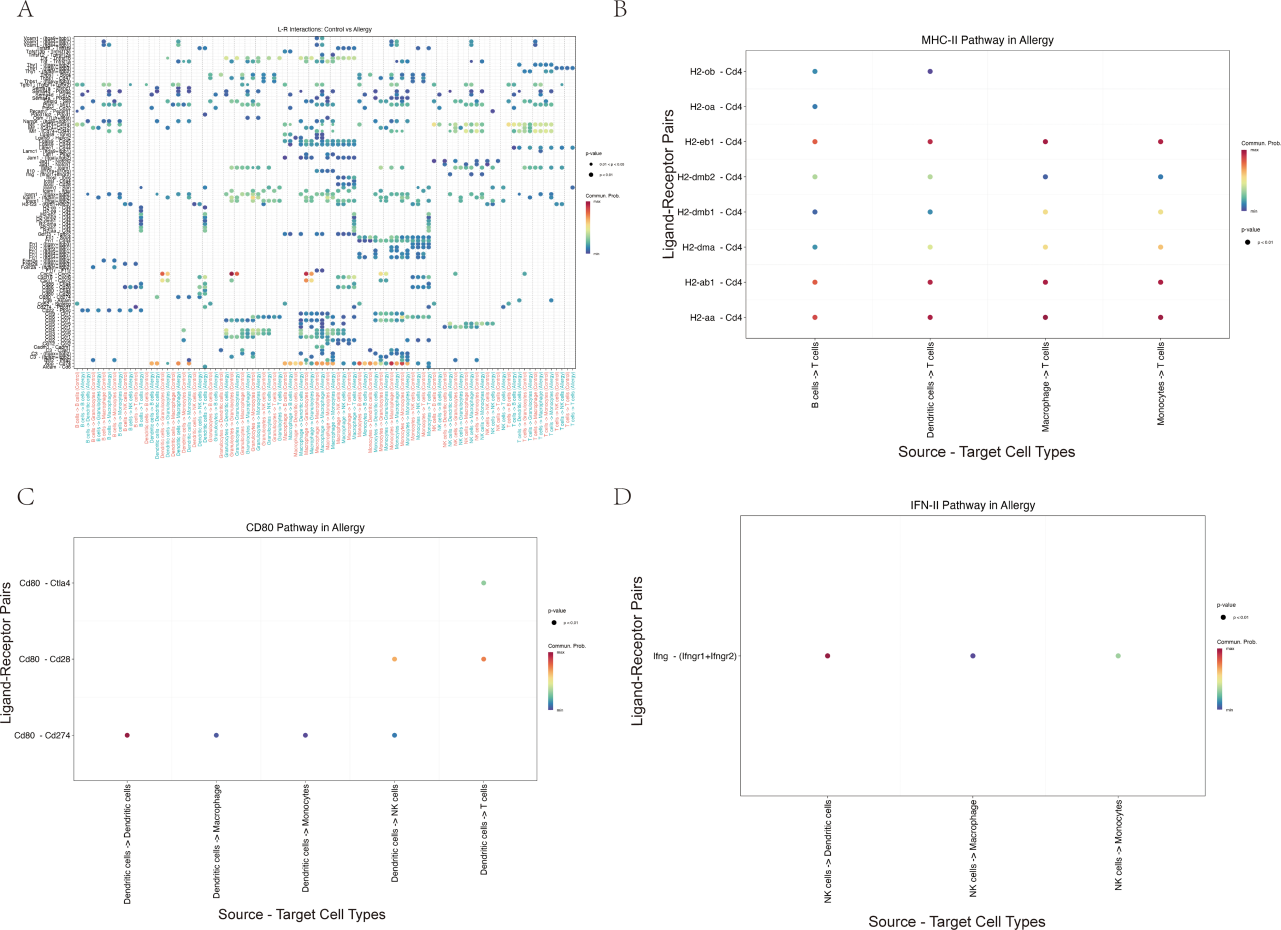
Cell–cell communication analysis suggests a remodeled immune network in allergic conjunctivitis, with enhanced inferred APC–T cell and NK–APC signaling. **(A)** Hierarchical plot providing a global overview of the strength of significant inferred ligand–receptor interactions between the major conjunctival cell types. Rows represent signaling pathways and columns represent cell–cell interaction pairs. **(B–D)** Dot plots detailing specific ligand–receptor interactions within key immune pathways inferred to be increased in the Allergy group. In these plots, dot color indicates the communication probability and dot size reflects statistical significance. **(B)** Analysis of the MHC-II pathway indicates stronger inferred antigen presentation signaling from antigen-presenting cells (APCs) to T cells. **(C)** Analysis of the CD80 pathway suggests increased co-stimulatory signaling, primarily from dendritic cells (DCs) to T cells and NK cells. **(D)** Analysis of the IFN-II (interferon-γ) pathway highlights an inferred communication pattern in which NK cells act as a major source of IFN-γ signals directed towards APCs.

Cell–cell communication analysis using CellChat revealed a markedly remodeled immune interaction network in AC (**Figure 9A**). Compared with controls, antigen-presenting cells (APCs), including macrophages and dendritic cells, displayed stronger inferred MHC-II and CD80 signaling to T cells (**Figures 9B, C**). In parallel, NK cells emerged as a major inferred source of IFN-γ signaling, predominantly targeting APCs (**Figure 9D**). Taken together, these correlative data are consistent with a model in which an IL-18R/IFN-γ axis in NK and T cells is linked to enhanced APC–T cell crosstalk and a more activated inflammatory network in AC, although functional experiments will be required to establish causality.

### 3.4 NK and T cells exhibit distinct NF-κB and JAK–STAT activation trajectories

**Figure 10:**
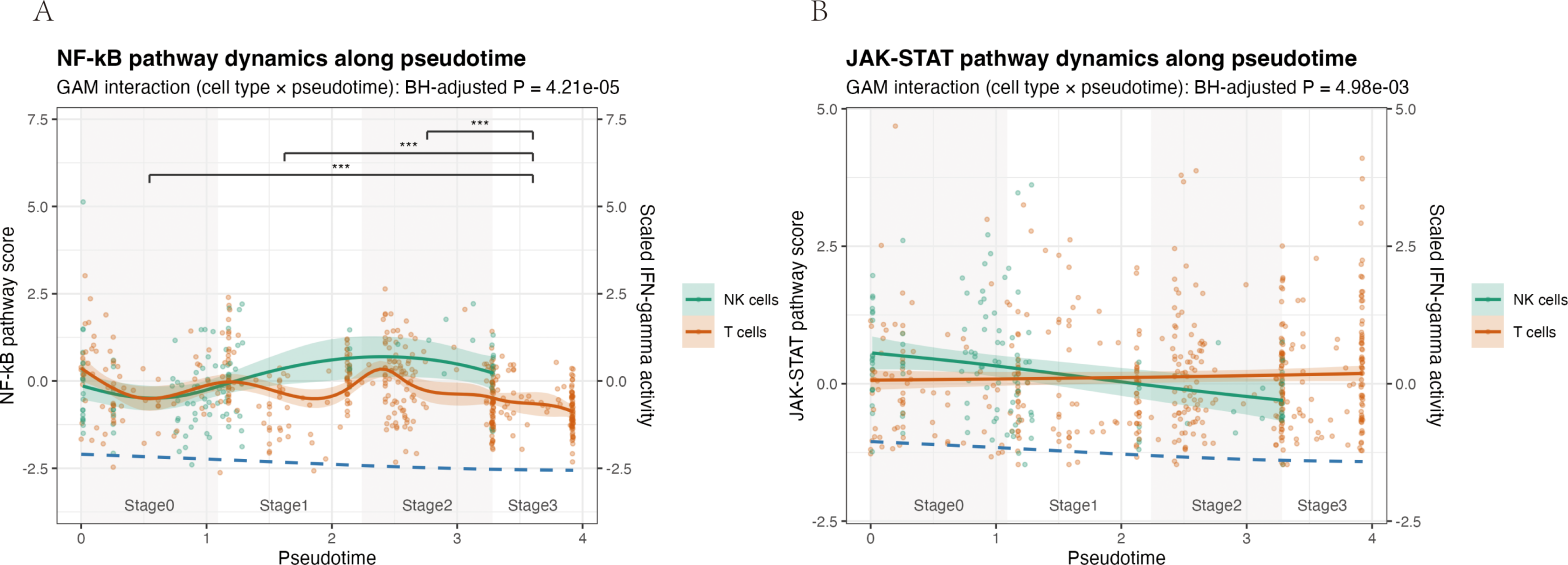
Pseudotime analysis reveals distinct activation dynamics of NF-κB and JAK–STAT pathways in NK and T cells. Pathway activity scores were modelled along a pseudotime trajectory for NK cells (teal) and T cells (orange) isolated from the allergic conjunctivitis mouse model. Solid lines show generalized additive model (GAM) fits, with shaded areas indicating the 95% confidence interval; dots represent individual cells. A scaled IFN-γ pathway activity score is overlaid as a dashed blue line for temporal reference. **(A)** NF-κB pathway. The interaction between cell type and pseudotime was significant (GAM interaction, Benjamini–Hochberg–adjusted P = 4.21 × 10^-5^). NF-κB activity in NK cells rises from early pseudotime, reaches a clear maximum at intermediate stages (Stages 1–2), and declines at late pseudotime, whereas T cells display only modest fluctuations around baseline. **(B)** JAK–STAT pathway. A significant cell type × pseudotime interaction was also detected (GAM interaction, Benjamini–Hochberg–adjusted P = 4.98 × 10^-^³). JAK–STAT activity in NK cells decreases gradually along pseudotime, while remaining low and nearly flat in T cells.

Pseudotime trajectory analysis of NK and T cells revealed cell type–specific dynamics of NF-κB and JAK–STAT signaling (**Figure 10**). In NK cells, NF-κB pathway scores increased from early pseudotime, reached a clear maximum around intermediate stages (Stage1–2), and then declined sharply at late pseudotime (Stage3), whereas T cells showed only modest fluctuations around zero throughout the trajectory and remained consistently lower than NK cells. Accordingly, NF-κB activity differed significantly across pseudotime-defined stages (Kruskal–Wallis P = 1.4 × 10^-9^), driven primarily by a marked reduction at Stage3 compared with Stages0–2 (all pairwise Benjamini–Hochberg–adjusted P < 0.001). JAK–STAT scores in NK cells displayed a mild but continuous decrease along pseudotime, while T cells maintained low, nearly flat levels with only a slight upward trend. Generalized additive models confirmed significant pseudotime × cell-type interactions for both pathways (Benjamini–Hochberg–adjusted P = 4.2 × 10^-5^ for NF-κB and 5.0 × 10^-^³ for JAK–STAT), indicating that NK and T cells follow distinct temporal patterns of pathway engagement, with NK cells showing a more pronounced, transient activation profile and T cells exhibiting a comparatively muted, gradual modulation.

### 3.5 IL-18R–NF-κB signaling is activated *in vivo* in an OVA-induced AC model

In an OVA-induced AC mouse model, we reproduced key clinical and histological hallmarks of AC, including elevated serum IgE (P < 0.0001) and conjunctival eosinophil infiltration (**Figure 3**). In conjunctival tissue from allergic mice, mRNA levels of Il18r1 and Il18rap were significantly increased, accompanied by higher protein expression of IL18R1 and IL18RAP (qRT-PCR and Western blot, all P < 0.01) (**Figures 4A–E**). Phosphorylation of NF-κB p65, a marker of NF-κB activation, was also significantly elevated (P = 0.0206) (**Figure 4F**). These *in vivo* data confirm that the IL-18 receptor axis and downstream NF-κB signaling are upregulated and functionally engaged in AC.

### 3.6 IL-18 receptor axis expression increases with clinical severity of AC

To assess whether IL-18 receptor axis activation reflects disease severity, OVA-challenged mice were stratified into mild, moderate and severe groups according to clinical symptom scores. Conjunctival protein levels of IL-18, IL-18R1 and IL-18RAP increased stepwise from controls through mild, moderate and severe groups (**Figures 2A–D**). Ordinary one-way ANOVA showed highly significant differences among the four severity groups for all three proteins (all P < 0.0001). A test for linear trend across ordered severity categories demonstrated a strong severity-dependent rise in expression (IL-18: F = 533.6, *P* < 0.0001; IL-18R1: F = 291.7, *P* < 0.0001; IL-18RAP: F = 537.5, *P* < 0.0001). Thus, the IL-18 receptor axis is not only upregulated in AC but also scales with clinical severity at the group level.

## 4 Discussion

This study used an integrative strategy combining human multi-omics, a public HDM-induced AC single-cell dataset, and an independent OVA-induced AC mouse model with protein-level and severity-stratified validation to investigate genetic susceptibility and molecular mechanisms in AC. From large-scale human GWAS and TWAS, we identified nine AC-associated candidate genes. Mouse conjunctival scRNA-seq localized *Il18r1* and *Il18rap* upregulation to ocular surface NK and T cells and revealed remodeled immune communication and dynamic pathway activity. In parallel, experiments in the OVA-induced AC model confirmed increased conjunctival IL-18 receptor axis proteins and NF-κB p65 phosphorylation and showed that IL-18, IL-18R1 and IL-18RAP expression increases in parallel with clinical severity. Together, these convergent data suggest that genetic variation in the IL-1 receptor family, particularly the IL-18 receptor components, is associated with a cross-species signature of immune activation in AC.

### 4.1 Interpretation of key findings in AC pathogenesis

First, human genetic and transcriptomic data provide the population-level starting point of the stepwise pipeline, which moves from human population genetics to cellular and tissue-level readouts in experimental models. In the human layer, multi-omics integration (FUSION, MAGMA and UTMOST) across large GWAS datasets highlighted nine AC-related genes, with a prominent signal at the *IL18R1–IL18RAP* locus.

Second, mouse conjunctival scRNA-seq in the HDM-induced AC model showed that *Il18r1* and *Il18rap* transcripts are selectively upregulated in ocular surface NK and T cells, suggesting heightened responsiveness of these lymphocyte populations to IL-18. Cell–cell communication analysis further indicated increased MHC-II antigen presentation and CD80 co-stimulation in allergic mice, along with enhanced NK-cell-dominated IFN-II (IFN-γ) signaling targeting antigen-presenting cells (APCs). Pseudotime modelling further showed that NF-κB and JAK–STAT pathways follow distinct, cell type–specific temporal trajectories, with NK cells exhibiting an early, high-amplitude response whereas T cells display a lower, more sustained activation profile.

Third, protein-level and severity-stratified validation in the OVA model demonstrated increased conjunctival IL-18R1 and IL-18RAP expression and elevated NF-κB p65 phosphorylation in OVA-challenged versus control mice, supporting the inference that these pathways are not only transcriptionally engaged but also biochemically active *in vivo*. When OVA-challenged mice were stratified into mild, moderate and severe groups based on clinical symptom scores, conjunctival IL-18, IL-18R1 and IL-18RAP protein levels showed a stepwise increase from controls through the three severity categories. Ordinary one-way ANOVA and tests for linear trend confirmed a robust severity-dependent gradient for all three proteins. These data indicate that, in this model, the IL-18 receptor axis is not simply on or off in AC but shows a group-level association between the magnitude of its activation and clinical severity. Because the data are cross-sectional and derived from group comparisons, they should be interpreted as associative rather than as proof that IL-18 receptor activation determines disease severity at the individual level.

The classical AC framework emphasizes IgE-mediated type I hypersensitivity and the Th2–mast cell–eosinophil axis. While this paradigm explains many clinical and pathological features, it does not fully account for the heterogeneity of AC, including subtype differences (e.g. SAC, PAC, VKC and AKC) and treatment-refractory disease. The present multi-layered findings suggest that, in addition to Th2 responses, NK cell activation, an IL-18R/IFN-γ axis, and widespread NF-κB and JAK–STAT pathway engagement are associated with AC, thereby broadening the immunoregulatory landscape. IL-18 has been reported to be elevated in allergic diseases and to promote mast cell activation, eosinophil recruitment and IgE production in experimental systems (35). The high IL-18R expression on NK and T cells observed in the AC mouse conjunctiva is compatible with a model in which IL-18-responsive lymphocytes contribute to amplifying local inflammation. This multi-pathway, multi-cell involvement offers a plausible explanation for the clinical diversity and persistence of AC but does not, by itself, establish mechanistic causality.

### 4.2 Novel genetic insights into AC: the 9 consensus genes and their potential roles

Integration of FUSION, MAGMA, and UTMOST on large-scale GWAS data identified nine consensus AC candidate genes: *IL1RL2*, *IL1RL1*, *IL18R1*, *IL18RAP*, *GAL3ST2*, *TLR10*, *TLR6*, *BBS12*, and *FAM114A1*. Among these, the *IL1RL1–IL18R1–IL18RAP* cluster on chromosome 2 is prominent: the *IL18R1/IL18RAP* region carries the strongest association in FinnGen R11 (lead variant rs72823641, P = 4.11 × 10^-^¹^8^) and forms a single high-LD block encompassing both genes, in line with prior reports implicating this region in ocular immune disease (36). In our regional association plot (**Figure 5B**), genome-wide significant variants form one broad, continuous peak from *IL1RL1* through *IL18R1* to *IL18RAP*, with *IL1RL1*-proximal variants largely in high LD with rs72823641, arguing against a clearly independent *IL1RL1* peak. This pattern suggests a shared association signal across the *IL1RL1–IL18R1–IL18RAP* cluster and supports a central role of the IL-18 receptor axis in AC.

Within this cluster, *IL1RL1*, *IL1RL2*, *IL18R1* and *IL18RAP* highlight the contribution of the IL-1 receptor family. *IL18R1* and *IL18RAP* encode the IL-18 receptor subunits IL-18Rα and IL-18Rβ. Their upregulation in NK and T cells in the AC mouse model correlated with enhanced IFN-γ signaling, consistent with a model in which IL-18 signaling contributes to IFN-γ production and with previous reports implicating IL-18 in allergic and autoimmune diseases (34). *IL1RL1* (ST2) encodes the IL-33 receptor, and the IL-33/ST2 axis is a well-established driver of type 2 immune responses and allergic diseases, including AC (37).

The inclusion of *TLR6* and *TLR10* points to a complementary role of innate immune recognition in AC pathogenesis (38). As pattern-recognition receptors, TLRs initiate signaling cascades that shape adaptive responses, including Th2 cell differentiation, which is highly relevant on the environmentally exposed ocular surface. Finally, *GAL3ST2*, *BBS12* and *FAM114A1* extend the pathogenic framework beyond canonical immune receptors. *GAL3ST2* encodes galactose-3-O-sulfotransferase 2, regulating glycan sulfation and thereby modulating cell adhesion, receptor–ligand interactions and potentially mucin properties at the ocular surface (39). *BBS12*, a Bardet–Biedl syndrome gene, is required for primary cilia formation and function (40); ciliary dysfunction could disturb ocular surface homeostasis and immune signaling. *FAM114A1* is less well characterized but has been linked to neuronal development, podocyte cytoskeleton maintenance and possibly B-cell function, with emerging data suggesting associations with allergic disease, potentially via angiotensin II–related pathways (41). In this study, these three genes are supported mainly by human genetic evidence; dedicated functional studies in ocular tissues or AC models will be needed to determine whether they act as causal drivers or markers of broader regulatory programs.

### 4.3 The IL-18R/IFN-γ axis as a novel pathway in AC

Across the different data layers, the IL-18R and IFN-γ axis emerges as a recurrent theme rather than from a single dataset. In human cohorts, IL18R1 and IL18RAP show strong, concordant association signals with AC. In the mouse model, conjunctival scRNA-seq demonstrates that *Il18r1* and *Il18rap* are upregulated in NK and T cells, and that their expression levels correlate with *Ifng* in allergic animals. Cell communication analysis indicates that IFN-II (i.e. IFN-γ–mediated) signaling from NK cells is enhanced in AC and is preferentially directed toward dendritic cells and macrophages, suggesting an activated NK–APC axis in the allergic state. At the protein level, conjunctival IL-18, IL-18R1 and IL-18RAP concentrations are higher in OVA-treated mice than in controls and display a clear stepwise increase from mild to moderate to severe disease categories.

This cross-layer consistency—from human genetic signals to mouse cell-type-specific expression and then to protein abundance and severity-stratified readouts—supports the interpretation that the IL-18 receptor axis is involved in AC pathophysiology and is associated with disease burden in this model. NK cells are important innate lymphocytes with cytotoxic functions and the capacity to produce cytokines such as IFN-γ. Increased NK cell numbers and activity have been reported in severe AC subtypes, including VKC (42). IFN-γ’s role in allergy is context-dependent. Although traditionally viewed as antagonistic to Th2 responses, several studies show that IFN-γ can also participate in, or amplify, ocular surface inflammation in severe allergic conditions (12). In active VKC, both Th1 (IFN-γ) and Th2 cytokines are markedly elevated. The enhanced IFN-γ signaling observed here, downstream of an IL-18-responsive NK and T-cell compartment, is compatible with a scenario in which IL-18 and IFN-γ crosstalk contributes to sustaining conjunctival inflammation, for example by modulating APC activation, leukocyte recruitment or structural cell responses. The linear trend in IL-18, IL-18R1 and IL-18RAP levels across ordered severity categories is in line with the idea of an amplification loop. However, because these data are correlative and cross-sectional, and because IL-18 and IFN-γ have pleiotropic effects, they do not resolve whether IL-18R and IFN-γ activity predominantly initiates, perpetuates or merely reflects more intense inflammation.

### 4.4 Remodeled immune cell communication network in allergic conjunctivitis

Cell-cell interactions are central to the initiation and regulation of immune responses (43). By applying CellChat to conjunctival scRNA-seq data, we observed substantial remodeling of the intercellular communication network in AC compared with controls. Both the number and overall strength of inferred signaling interactions were increased in the allergic group, particularly in pathways linked to antigen presentation (MHC-II) and T cell co-stimulation (CD80) (44). These findings suggest that AC is associated with a rewiring of immune communication toward more densely connected, pro-inflammatory circuits.

Within this network, APCs (dendritic cells and macrophages) and NK and T cells appear as important hubs, engaging in enhanced MHC-II and CD80 mediated interactions. This organization is consistent with a scenario in which antigen presentation and co-stimulation are upregulated during allergic responses, potentially facilitating the activation and maintenance of effector lymphocytes. The scRNA-seq-based communication inferences are model-based and do not directly measure ligand-receptor engagement or downstream signaling events. Nonetheless, the convergence of these computational predictions with the observed upregulation of IL-18R and IFN-γ related genes and NF-κB and JAK-STAT pathway signatures supports the view that AC involves a broadly sensitized immune network rather than isolated cell-type activation.

### 4.5 NF-κB and JAK-STAT pathway dynamics in AC

Pseudotime analysis of conjunctival NK and T cells in the HDM-induced AC model showed that NF-κB and JAK–STAT signaling are not uniformly upregulated but follow distinct, cell type–specific activation trajectories along pseudotime. In NK cells, NF-κB pathway scores were low at the beginning of the trajectory, rose sharply to a prominent peak at early to mid pseudotime and then declined, whereas JAK–STAT activity was highest at the start and progressively decreased thereafter. By contrast, T cells displayed only a modest, earlier NF-κB increase and maintained relatively low and stable JAK–STAT scores across pseudotime. These patterns, which were supported by significant pseudotime × cell-type interactions in the GAM models, are consistent with a scenario in which IL-18R–high NK cells contribute to an early, high-amplitude innate IFN-γ–linked burst of NF-κB and JAK–STAT activation, while T cells sustain a lower-grade adaptive program rather than simply mirroring the NK response (45, 46).

### 4.6 Study strengths, limitations, and considerations

A major strength of this study is the explicit linkage of human genetic data to cellular and molecular phenotypes in a controlled animal model, including both transcriptomic and protein-level validation. The multi-omics workflow, moving from population-level susceptibility signals to single-cell resolved immune landscapes and finally to biochemical and clinical readouts, provides a coherent framework for interpreting how AC risk loci may map onto conjunctival immune biology.

Several limitations should be considered when interpreting these results. First, functional validation of seven of the nine candidate genes (*IL1RL2, IL1RL1, GAL3ST2, TLR10, TLR6, BBS12* and *FAM114A1*) in ocular tissues or AC models is lacking; their roles remain inferential and based mainly on association and prior literature. Second, the OVA-induced mouse model recapitulates many features of acute AC but does not fully reflect the chronic, relapsing and sometimes scarring phenotypes observed in human disease (47). Third, the scRNA-seq-based inferences of cell-cell communication and pathway activity rely on computational frameworks and require direct experimental confirmation, for example through functional blocking of specific ligand-receptor interactions or signaling nodes. Fourth, our analyses focus primarily on immune cells and do not adequately capture the contributions of resident structural cells, such as epithelial, goblet and stromal cells, which may play important roles in barrier function and cytokine production.

Importantly, the severity-stratified analysis was performed in a relatively small cohort of mice, using ordinal categories based on clinical scores. As a result, the linear trends observed for conjunctival IL-18, IL-18R1 and IL-18RAP should be viewed as group-level associations rather than robust predictors of individual severity. These data support the concept that greater activation of the IL-18 receptor axis accompanies more severe clinical disease in this model but do not prove that the axis itself determines severity. Genetic risk interactions with environmental exposures, such as microbial patterns or aeroallergens, also remain unexplored. Finally, Mendelian randomization analyses were not conducted. Consequently, the current study does not provide formal evidence of causality between the identified genetic variants, IL-18R and IFN-γ signaling and AC risk.

## Data Availability

The processed single-cell RNA-seq Seurat object and analysis scripts have been deposited in Figshare (https://doi.org/10.6084/m9.figshare.30633122). The dataset is publicly available and searchable.
